# Agricultural SandboxNL: A national-scale database of parcel-level processed Sentinel-1 SAR data

**DOI:** 10.1038/s41597-022-01474-4

**Published:** 2022-07-13

**Authors:** Vineet Kumar, Manuel Huber, Björn Rommen, Susan C. Steele-Dunne

**Affiliations:** 1 Department of Geoscience and Remote Sensing, Delft University of Technology, Delft, 2600AA The Netherlands; 2 Department of Water Management, Delft University of Technology, Delft, 2600AA The Netherlands; 3 European Space Agency (ESA/ESTEC), Noordwijk, 2201AZ The Netherlands

**Keywords:** Agriculture, Scientific community

## Abstract

Synthetic Aperture Radar (SAR) data handling, processing, and interpretation are barriers preventing a rapid uptake of SAR data by application specialists and non-expert domain users in the field of agricultural monitoring. To improve the accessibility of Sentinel-1 data, we have generated a reduced-volume, multi-year Sentinel-1 SAR database. It includes mean and standard deviation of VV, VH and VH/VV backscatter, pixel counts, geometry, crop type, local incidence angle and azimuth angle at parcel-level. The database uses around 3100 Sentinel-1 images (5 TB) to produce a 12 GB time series database for approximately 770,000 crop parcels over the Netherlands for a period of three years. The database can be queried by Sentinel-1 system parameters (e.g. relative orbit) or user application-specific parameters (e.g. crop type, spatial extent, time period) for parcel level assessment. The database can be used to accelerate the development of new tools, applications and methodologies for agricultural and water related applications, such as parcel-level crop bio-geophysical parameter estimation, inter-annual variability analysis, drought monitoring, grassland monitoring and agricultural management decision-support.

## Abstract (summary)

**Table:** 

Measurement(s)	parcel-level backscatter • cross-ratio
Technology Type(s)	SAR remote sensing • Sentinel-1 • Google Earth Engine
Sample Characteristic - Location	The Netherlands

## Background & Summary

Synthetic Aperture Radar (SAR) satellite sensors provide Earth Observation (EO) data without being hindered by weather or solar illumination. The interaction of radar (microwave) signals with the surface depends on the properties of radar system as well as the target characteristics. In particular, changes in the geometric and dielectric properties of targets are associated with the backscattered signal received by a SAR system for a given resolution cell^1^.

The Copernicus Sentinel-1 SAR mission is a joint initiative of the European Space Agency (ESA) and the European Commission (EC) launched to image the global landmass, coastal zones, sea ice and polar areas at high geometric and radiometric resolutions. The mission comprising two identical satellites, Sentinel-1A (launched 3 April 2014) and Sentinel-1B (launched 25 April 2016) offers 6 day repeat cycle and acquires data at 5.405 GHz (C-band)^2^. The operational interferometric wide (IW) mode provides dual-pol (VV + VH or HH + HV) data for a 250 km swath, with incidence angle variations from 30° to 46°. By combining multiple orbital tracks from ascending and descending passes, it is possible to obtain Sentinel-1 data every 1–2 day in Europe^3^.

The large data volumes associated with SAR data create a need for high performance computing and data storage facilities for service providers as well as data users. For example, typical unzipped Sentinel-1 IW Single Look Complex (SLC), and Ground Range Detected (GRD) products (250 km by 175 km) have data volumes of around 7 GB and 1.6 GB respectively. Recently, it has been reported that around 6 million Sentinel-1 data products, with a data volume of 10 petabytes were generated by the end of 2020^4^.

Due to these large data volumes, conventional methods of satellite data distribution, download, storing and processing may pose serious challenges to users who want to process imagery with local computing resources^5^. Regional and national-level monitoring applications such as agriculture, forestry, wetlands, urban area and infrastructure monitoring where past, requiring present and future dense time-series of SAR data necessitate the use of distributed computing platforms with integrated data repositories.

Cloud computing infrastructures such as Google Earth Engine (GEE) and Amazon Web Services (AWS) hosting the pre-processed historical and recently acquired satellite datasets^6–8^ have been enabled to ingest (amongst others) Copernicus Sentinel datasets allowing ease of data access and further processing. GEE is a cloud-based platform that archives a range of openly-available geospatial data and provides users access to high performance parallel computing with an internet-based application programming interface (API). GEE contains several satellite, thematic, vector, demographic and climate datasets^9^. The computing capabilities of GEE obviate the need for users to download, store and process high volume spatio-temporal data and ensure that this can be done in a fast and efficient manner^10^. The utility of the GEE platform has been shown in many studies involving large scale land cover dynamics mapping^11,12^, crop assessment and classification at various scales^13–18^, yield mapping^19^, grassland monitoring^20^ global urban land mapping^21^, drought assessment^22^, snow depth variability^23^ and many others. A detailed review of GEE based publications for various thematic applications using GEE stored geo-spatial dataset has been summarized here^24–26^.

Spaceborne EO assets provide crucial information content for crop monitoring at regional, national and global scales. Information on parcel level crop dynamics is essential to support decision-making by farmers, agri-advisors, water boards and agro-farm industries. Advisors and grower organizations provide information to the farmers at parcel level. Parcel level spatio-temporal information from remote sensing observations facilitates the sustainable use of resources as well as precision farm management strategies^20,27–29^. Due to the burden of data storage and processing, SAR-based studies are typically limited to the smaller study areas. However, open availability of Sentinel-1 SAR GRD products in GEE enable to rapid production of parcel-level temporal signatures.

The aim of the Agricultural SandboxNL is to improve the accessibility of Sentinel-1 SAR data for application experts interested in adapting or developing tools to use Sentinel-1 for agricultural monitoring. The Netherlands is ideally suited to the development and testing of such methodologies. Geospatial data assets and statistics are openly available via *Publieke Dienstverlening Op de Kaart* (PDOK, Public Services on the Map)^30^ and Statistics Netherlands (CBS)^31^. In addition, satellite from commercial providers are acquired regularly, and made freely available via the Netherlands Satellite Data Portal^32^. The objective of this study is to produce an interactive database that contains information extracted from multi-temporal Sentinel-1 SAR datasets at parcel level. The output database will support a range of SAR data uses for operational purposes with a demonstration of spatially tagged parcel level backscatter scalable at various administrative levels. This will reduce the burden of processing and extraction of large volume of Sentinel-1 SAR data for expert and non-expert data users.

## Methods

**Fig. 1:**
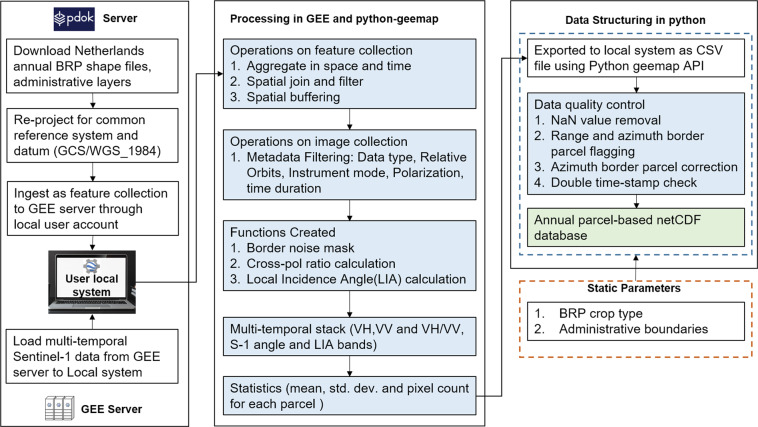
Proposed processing steps in the Agricultural SandboxNL database creation. The blue highlighted boxes indicate all processing steps applied onto the GRD data in order to create the database. The green box indicates the final dataset.

The methods section is divided into four subsections: (1) *Data sources* describes the source and characteristics of the input vector and SAR remote sensing datasets; (2) *Data processing* details the functionalities implemented in GEE for SAR and vector data aggregation and processing; (3) *Agricultural SandboxNL database generation* describes how the GEE processed Sentinel-1 GRD images were converted into an interactive user-ready agricultural crop parcel-level database; (4) *Sentinel-1 interferometric coherence* describes how SLC data were processed in SNAP and imported into GEE to generate a sample for the parcel-level database. The workflow adopted to generate the database is illustrated in Fig. 1.

### Data sources

#### Parcel data and attributes

A wide range of digital geospatial datasets in The Netherlands are openly accessed and hosted at the PDOK server under creative common licences (CC-BY-4.0). In Agricultural SandboxNL, annual *Basisregistratie Gewaspercelen* (BRP, Crop Parcel Base Register) and administrative layers such as province, municipality are used as base vector data^33^. The BRP dataset consists of the location of agricultural parcels, coupled with the cultivated crop name, unique parcel identifier and geometry. In total 312 distinct cultivated crops are defined in the BRP dataset. The boundaries of the agricultural parcels are based on *Agrarisch Areaal Nederland* (AAN, Agricultural Area of The Netherlands)^33^. The landholders of the parcels provide crop parcel boundaries every year, tagged with the main cultivated crop name. This information exchange is required by Dutch law and guarantees annually updated national crop information. Since 2009, a draft data set with the reference date of 15 May is generated for each year and uploaded annually at PDOK web server. In this work, BRP vector data for the years 2017, 2018 and 2019 were used to provide parcel boundaries and local (parcel-level) crop type information.

#### Sentinel-1 SAR data

**Fig. 2:**
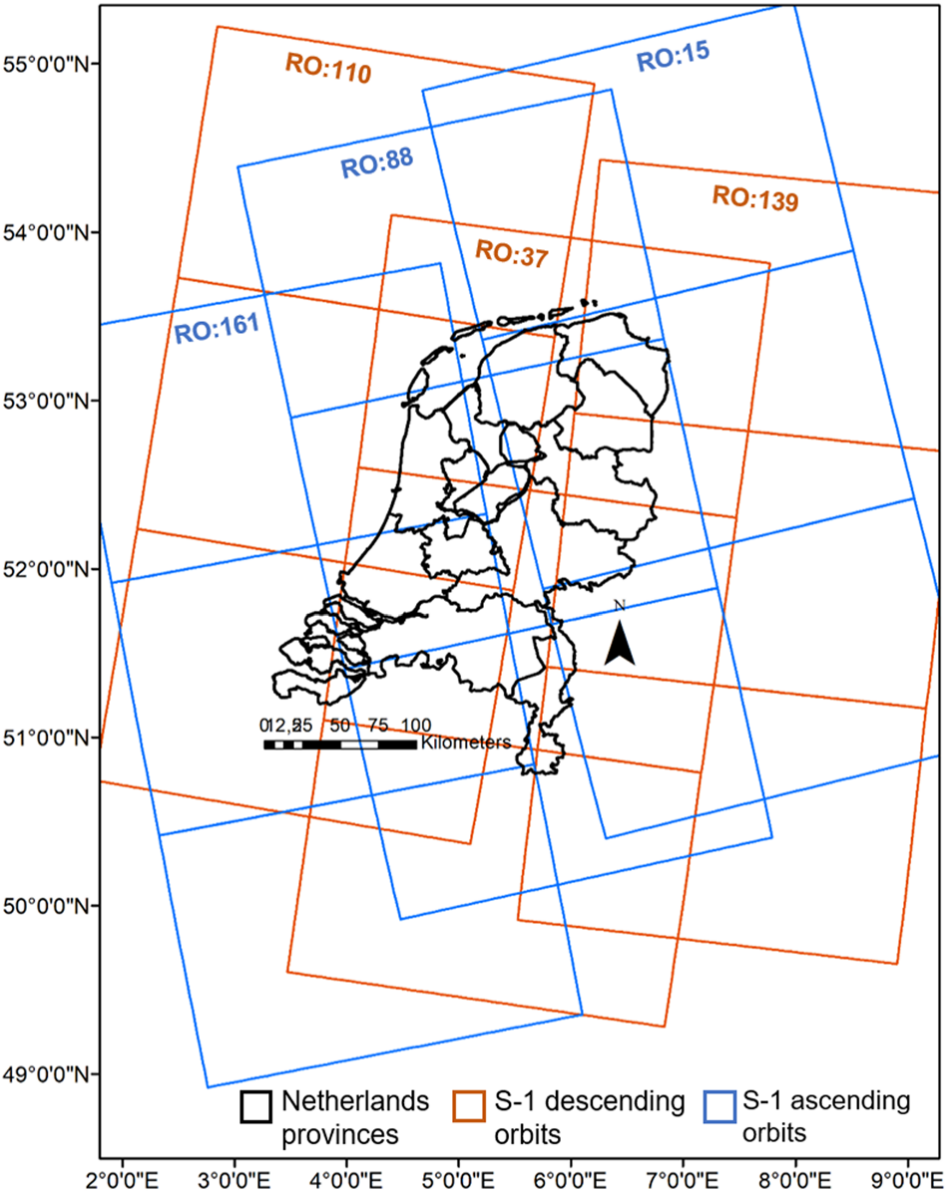
Sentinel-1 coverage over The Netherlands based on six relative orbits (RO). The blue polygons indicate ascending orbits (161, 88, 15) and the orange indicate descending orbits (139, 110, 37).

GEE contains calibrated and ortho-rectified GRD products of Sentinel-1 SAR data, details of which are provided in Table 1. GEE-ingested Sentinel-1 data is pre-processed in the Sentinel Application Platform (SNAP) SAR data processing toolbox^34^ using the following steps: (1) Metadata update using restituted orbit files (2) Border noise removal (3) Thermal Noise Removal (4) Radiometric calibration (5) Terrain correction using Shuttle Radar Topography Mission (SRTM) 30 m or Advanced Spaceborne Thermal Emission and Reflection Radiometer (ASTER) Digital Elevation Model (DEM)^35^. The final terrain-corrected Sigma Nought *σ*° backscatter values are available to fetch on a linear or logarithmic (dB) scale. All images also include an additional ‘angle’ band that contains the approximate viewing incidence angle. SRTM 30 meter v3 DEM product provided by NASA was used as an auxiliary data to generate the azimuth and local incidence angle band for Sentinel-1 SAR images. In GEE, Sentinel-1 GRD products are available over the globe since October 2014. The current version of the Agricultural SandboxNL database includes three years of Sentinel-1A/B images. Data from 2017 onwards were used due to the higher temporal resolution following the launch of Sentinel-1B in 2016. Sentinel-1 passes in 8 different relative orbits (RO) over the Netherlands in ascending (evening) and descending (morning) tracks. However, 6 RO (Ascending (88, 161, 15) and Descending (37, 110, 139)) cover almost the entire geographical extent of the Netherlands (Fig. 2), so these were used here.

**Table 1:** Specification of Sentinel-1 SAR data hosted at GEE platform.

Sentinel-1	Data specifications in GEE
Product type	Ground Range Detected (GRD)
Data availability	Since April 2014
Acquisition mode	IW (Interferometric Wide), EW (Extra Wide) and SM (Strip Map)
Polarizations	VV, HH, VV + VH and HH + HV
Spatial resolution (posting)	10, 25 and 40 meter
Thermal noise removal	After July 2015 acquisitions
Border noise removal	After March 2018 acquisitions

### Data processing

#### Vector data processing

**Fig. 3:**
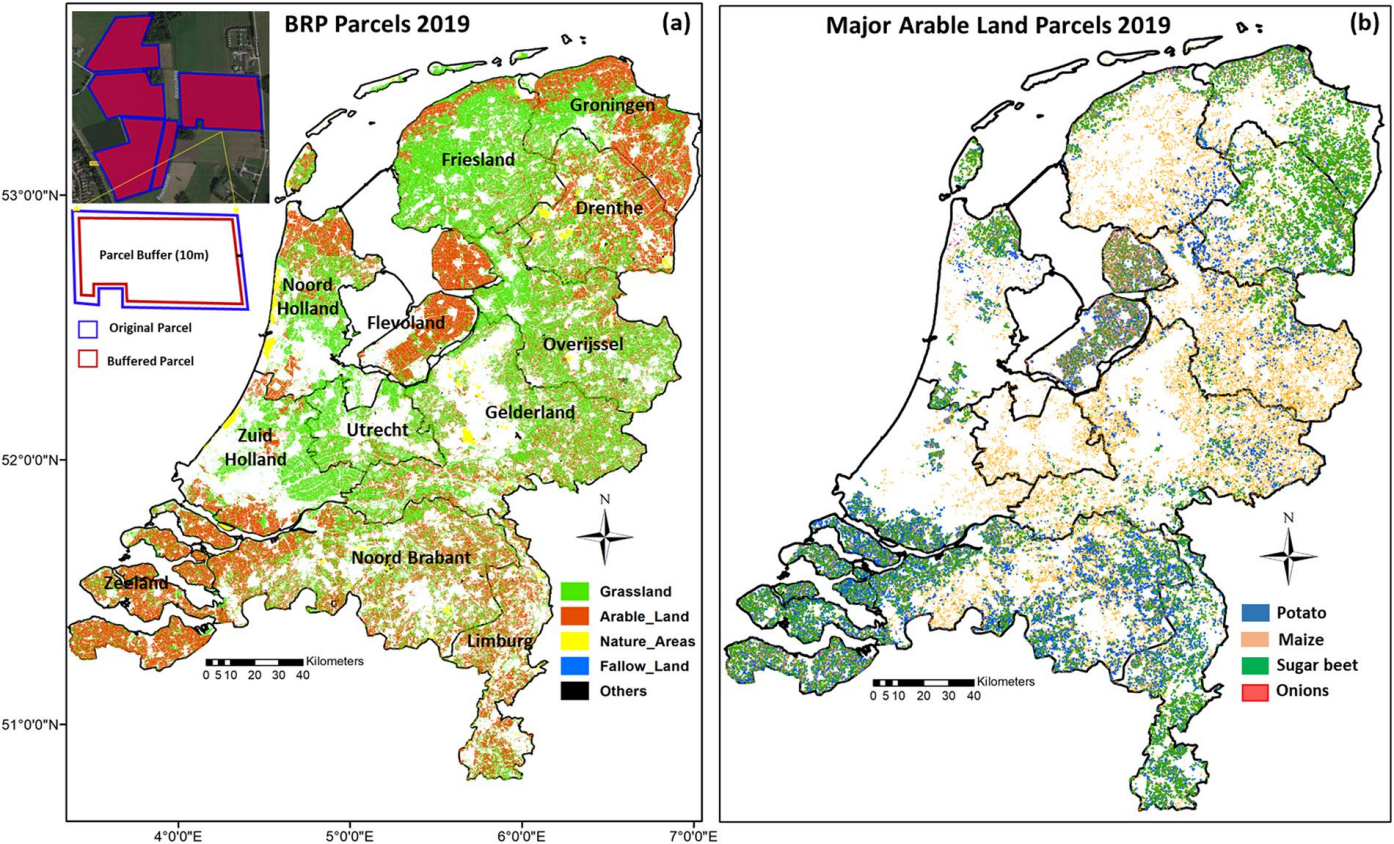
Two example images illustrating the BRP vector data for major crops of The Netherlands. The left image (**a**) indicates crop classes such as grassland, arable land, nature areas and fallow land, whereas the right image (**b**) shows the major arable crop types (potato, maize, sugar beet and onions). The inset image in the upper-left corner of the left image shows the 10 m spatial buffer applied on each parcel.

The downloaded BRP vector layers from the PDOK server has an Amersfoort/RD New projected coordinate system (EPSG = 28992), so those needed to be converted to geographical coordinates (EPSG: 4326) to be imported as a vector data asset in GEE platform. A spatial buffer of −10 m was applied to the parcel polygons in the BRP data to remove edge effects due to e.g. parcel ridges, water channels, roads, building shades, adjacent fields. This is illustrated in Fig. 3(a). Parcels smaller than 100*m*^2^ were excluded from the database as the application of the buffer would make them prohibitively small. The centroid of each buffered parcel was calculated in GEE and added as an extra feature property to the BRP vector data to prevent ambiguity when parcels span administrative boundaries.

Figure 3(a) shows all 772,565 BRP parcels classified into 5 major land cover classes for the year 2019. It indicates that grassland is the dominant cover type, accounting for 66% of all parcels. Figure 3(b) shows the spatial distribution of the main summer crops in the Netherlands, namely silage maize, consumption potato, sugar beet and sow onions. Collectively, these crops account for 110,000 parcels.

#### Sentinel-1 SAR data processing

A set of processing steps were applied to the Sentinel-1 data in GEE before extracting the values for the database. The metadata of Sentinel-1 data at linear scale *S1_GRD_FLOAT* were filtered for system parameters such as IW acquisition mode, overpass type (ascending/descending), 6 different RO and dual (VV + VH) polarization in space by using administrative boundaries and in time for each 2017, 2018 and 2019 year separately. Next, the following functions were executed in GEE on the filtered Sentinel-1 image collections.

#### Border noise removal

**Fig. 4:**
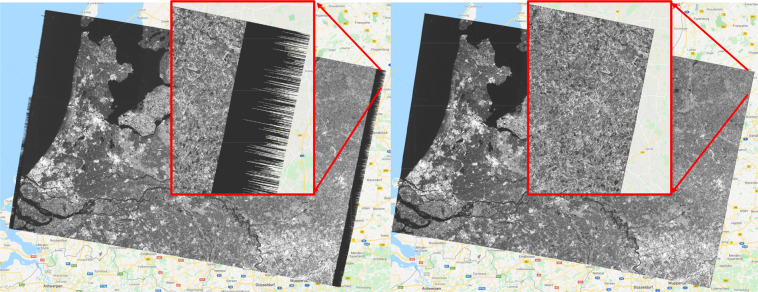
Border noise removal example for January 2018, orbit 37 from Sentinel 1 A GRD (VH). The left image shows an image before border noise masking and the right image shows the image after masking.

The transformation of Sentinel-1 level-0 to level-1 products using the ESA Instrument Processing Facility (IPF)^36^ leads to generation of artifacts such as “no-value” and “very low values” pixels due to azimuth/range compression and the sampling start time window offsets^5^ at the range and azimuth borders of the image data. The effect of border noise pixels has been resolved in IPF v 2.90 and later versions (after March 2018 products in GEE^35^). However, for earlier IPF versions, these effects can be observed at the borders of GRD data products (Fig. 4). In the Agricultural SandboxNL, imagery from 2017, 2018 and 2019 were considered for database generation. Hence, removal of these “no-value” and “very low values” border pixels was an important step to ensure that the data record is consistent for multi-year studies. Outside the GEE platform, the SNAP toolbox provides an operator *Sentinel-1 Remove GRD border noise* that uses de-noising vectors on the VV or HH-pol data by first setting dark pixels to zero values and then removing them using a thresholding approach^36^. Several approaches, namely anomaly detection based on statistical interquartile range^5^, bidirectional sampling based^5^ and mathematical morphology^37^ based methods are introduced in the literature. However, their implementation in GEE is more challenging due to their complexity and computational efficiency for large volume of data. Within the GEE environment, previous studies have applied single value backscatter thresholds^38^, and median composite filter^39^ on the entire image. However, these might affect other pixel values in addition to the border pixels.

To overcome the border noise issue in the Agricultural SandboxNL dataset, we have employed a simple threshold technique on the outer edges (near and far range) of the image. This means the threshold was only applied on pixels with an approximate viewing incidence angle of <30 degrees and >45 degrees. To remove pixels with very low values a threshold value of 0.003 linear or −35 dB was applied. Other advantages of this simple technique are the simple implementation and the computational efficiency, as it was only applied to the image border regions. This mask was created using VV polarization values only, and applied over the image collection for both VV and VH pol data. This avoids any incorrect removal of low pixel values in cross-polarization. To have consistent results, the masking was executed for all images, including 2019. As the approach was based on simple value masking, there are chances that some *NaN* or very low data values remain present at border pixels. Those *NaN* pixels were removed in the later steps of database creation. An example illustrating the effect of border noise removal on January 2018 (RO-37) is shown in Fig. 4.

#### Local incidence angle calculation

Radar backscatter depends on both system and target characteristics. Due to the influence of surface geometry on radar backscatter, local incidence angle (LIA) needs to be taken into account for the retrieval of geophysical variables of interest^40^. Calculation of local incidence angle requires geometric information from the viewing radar and the terrain^40^. Terrain geometry parameters, namely slope and aspect values, were extracted from the SRTM30 DEM which is available in GEE. Radar look direction is defined by the incidence angle and range-look direction. In this study, approximate viewing incidence angle information was directly used as captured by Sentinel-1 GRD data in GEE^41^. However, range direction information is not directly available with Sentinel-1 data products, hence it was calculated from Sentinel-1 image geometry by calculating azimuth information as discussed in^42,43^. Both LIA and azimuth angle were calculated separately for ascending and descending track images.

#### Sentinel-1 backscatter observable

Parcel-averaged backscatter coefficients in VV and VH are included in the Agricultural SandboxNL database. In addition, the parcel-averaged cross-polarization ratio (VH/VV) was calculated. The use of this ratio is becoming increasingly common in agricultural monitoring as it reduces the influence of soil moisture on the retrieval of vegetation parameters^44–47^. As shown in Fig. 2, the Netherlands is covered by six relative orbits of Sentinel-1 A/B, which results in different observation geometries and timestamps. Sentinel-1 backscatter (VV, VH and VH/VV) and associated attributes (LIA, azimuth angle and pixel counts) within the parcels were averaged and stored for each RO separately. This results in a table containing the mean and standard deviation of VV, VH and VH/VV, LIA, azimuth angle and pixel counts at parcel level with unique OBJECTIDs. Therefore, data from multiple relative orbits were never combined. The user can query and process the data using the relative orbit number. Note that a speckle noise removal filter was not implemented during the backscatter information extraction because the backscatter values were spatially averaged over the parcel. It is important to note that the database was generated using the GEE, based on the Sentinel-1 GRD data available on GEE. Terrain flattening is not available within GEE, and was therefore not applied in the current database. Although the Netherlands is generally relatively flat, users of the database should be mindful of this limitation. This is particularly relevant if users apply the code and/or methodology to other hilly or mountainous regions. In these regions, users are advised to use the terrain flattening module in SNAP to derive the terrain flattened *γ*0 ^48^, which is used as a standardized module in the Committee on Earth Observation Satellites Analysis Ready Data (COES ARD) initiative^49^. The LIA has been derived and was included in SandboxNL so that users can convert to e.g. *β*0 or *γ*0 products.

### Agricultural SandboxNL database generation

The border noise removal, local incidence angle determination and cross-ratio calculations were implemented in GEE environment. The standard programming language of GEE is *java* script. In this study the GEE Python API and additional python packages such as *geemap*^50^ were used to utilize the computational capability of GEE and its tools in a python environment. The *geemap* package, in particular, makes it possible to directly translate any given *java* script into python syntax.

The database generation involves several steps. First, all BRP and administrative boundary shapefiles needed to be uploaded manually as an asset in GEE whereas the elevation data (SRTM DEM) was directly imported from GEE. The second step was to control and manage the automation of the extraction process. This step poses several limitations and challenges. First, GEE has a memory threshold for each user to reduce overload of the system, which means only a selection of parcels can be processed and downloaded at a time. Another constraint was a discrepancy in GEE output values depending on the number and spatial extent of selected parcels. To circumvent these issues, the data were downloaded per municipality of The Netherlands to keep the feature collection extent relatively constant and localized. In addition, a maximum of 500 parcels were processed at a time to maintain a systematic mining process, and avoid memory limitations in the GEE. The border noise removal, cross-ratio determination and LIA calculation were applied for every batch of 500 parcels and the results were stored as a csv file on the local machine. The processing, flagging and cleaning of the data is explained in the *Data Records* section.

### Sentinel-1 Interferometric coherence

The primary dataset of the Agricultural SandboxNL is the Sentinel-1 radar backscatter because the value of backscatter has been widely demonstrated in mapping and monitoring activities. Several recent studies have highlighted the potential value of interferometric coherence for crop type mapping^51^, crop emergence, harvest detection^45,52^ and grassland monitoring^53^. However, GEE does not host the Sentinel-1 SLC products required to produce interferometric coherence^35^. In order to provide a sample of interferometric coherence data, the SLC images (RO 110) for the province of Flevoland were downloaded from the Copernicus Open Access Hub^54^ and processed offline in the SNAP toolbox for the 2019 growing season.

The coherence between two SLC images *S*1 and *S*2 is defined in Eq. 1. A standard processing chain^55^ was applied in the SNAP toolbox to calculate the 6-day InSAR coherence. The spatially-averaged parcel level interferometric coherence values were calculated from the 6-day pairs of Sentinel-1 in VV and VH polarizations. A total of 32 pairs of Sentinel-1 SLC products were analyzed during the period of April to October 2019. Data were processed for the province of Flevoland as the crop types in this area include structurally different crops such as potato, sugar beet, maize, and winter wheat. The SNAP generated coherence images in .*tiff* format were then ingested into GEE to create parcel-level data, in a format consistent with the backscatter data.

1 $$\gamma =\frac{| \langle {{\bf{S}}}_{1}{{\bf{S}}}_{2}^{* }\rangle | }{\sqrt{\langle {{\bf{S}}}_{1}{{\bf{S}}}_{1}^{* }\rangle \langle {{\bf{S}}}_{2}{{\bf{S}}}_{2}^{* }\rangle }}$$

## Data Records

The annual database consists of spatially averaged VV, VH and VH/VV backscatter values, corresponding standard deviation, viewing incidence angle, local incidence angle, relative orbit, azimuth angle and pixel count for each parcel. Every crop parcel has a unique field ID (OBJECTID), which is used as an index to sort and process the data. The data cleaning involves identification of parcels with no data due value due to their size smaller than 100 *m*^2^.

After cleaning the data, each parcel was stored separately in a data frame containing the time series of all the above-mentioned extracted parameters. A dictionary of all of these data frames was temporarily stored together with static information in form of a *pickle* file. A *pickle* file is a product of serializing a Python object structure into binary protocols using the pickle module in a python environment^56^. Static information includes attributes of the BRP vector data such as parcel geometry, area, centroid, crop type and flag information. Finally, the *pickle* dataset was transformed to a *netCDF* file to reduce the data size and to provide a universal data format for the scientific user community. Static information about the parcel geometry and crop type was not saved in the *netCDF* file but can be accessed via the BRP files. Table 2 gives an overview of variables stored in the final *netCDF* product. The datasets are provided per province and per year. A technical validation was conducted to flag all parcels which lie on the azimuth and range boundary of the image. Details about this validation and flagging process are discussed in the section “Technical Validation”.

**Table 2:** List of variables and their description in the SandboxNL *netCDF* product.

No.	Variable	Description
1	Time	Acquisition time of the SAR image
2	Longitude	Longitude of the parcel centroid
3	Latitude	Latitude of the parcel centroid
4	VV_mean	Average VV polarization backscatter intensity over parcel
5	VV_std	Standard deviation VV polarization
6	VH_mean	Average VH polarization backscatter intensity over parcel
7	VH_std	Standard deviation VH polarization backscatter over parcel
8	CR_mean	Average cross-pol ratio (VH/VV) over parcel
9	CR_std	Standard deviation cross-pol ratio (VH/VV) over parcel
10	LIA	Local incidence angle (adjusted for local topography)
11	EA	Approximate viewing incidence angle in the GEE S1 GRD product
12	AZA	Azimuth angle
13	OID	Object ID (unique per parcel)
14	MID	Sentinel-1 satellite mission ID (0 = S1A and 1 = S1B)
15	RO	Relative orbit tracks of Sentinel-1 used in database
16	Pix	Pixel count of the selected parcel
17	Flag	Flags assigned to border parcels for each relative orbit

The backscatter intensities of VV, VH and CR are stored in linear scale.

The Agricultural SandboxNL database supplemented by BRP parcel vector data is published for the public use, can be found on the 4TU Centre for Research Data repository (10.4121/14438750.v2)^57^. The readme files can be consulted for explanations of the data records, query and visualizations. Table 3 indicates the total no. of BRP parcels, Sentinel-1 GRD images processed and size of the output database annually.

**Table 3:** Details of Sentinel-1 images processed and output database size for a given year.

Year	BRP parcel counts	Sentinel-1 images processed	Output database size (GB)
2017	785710	1023	4.07
2018	774822	1030	3.99
2019	772565	1034	3.85

## Technical Validation

**Fig. 5:**
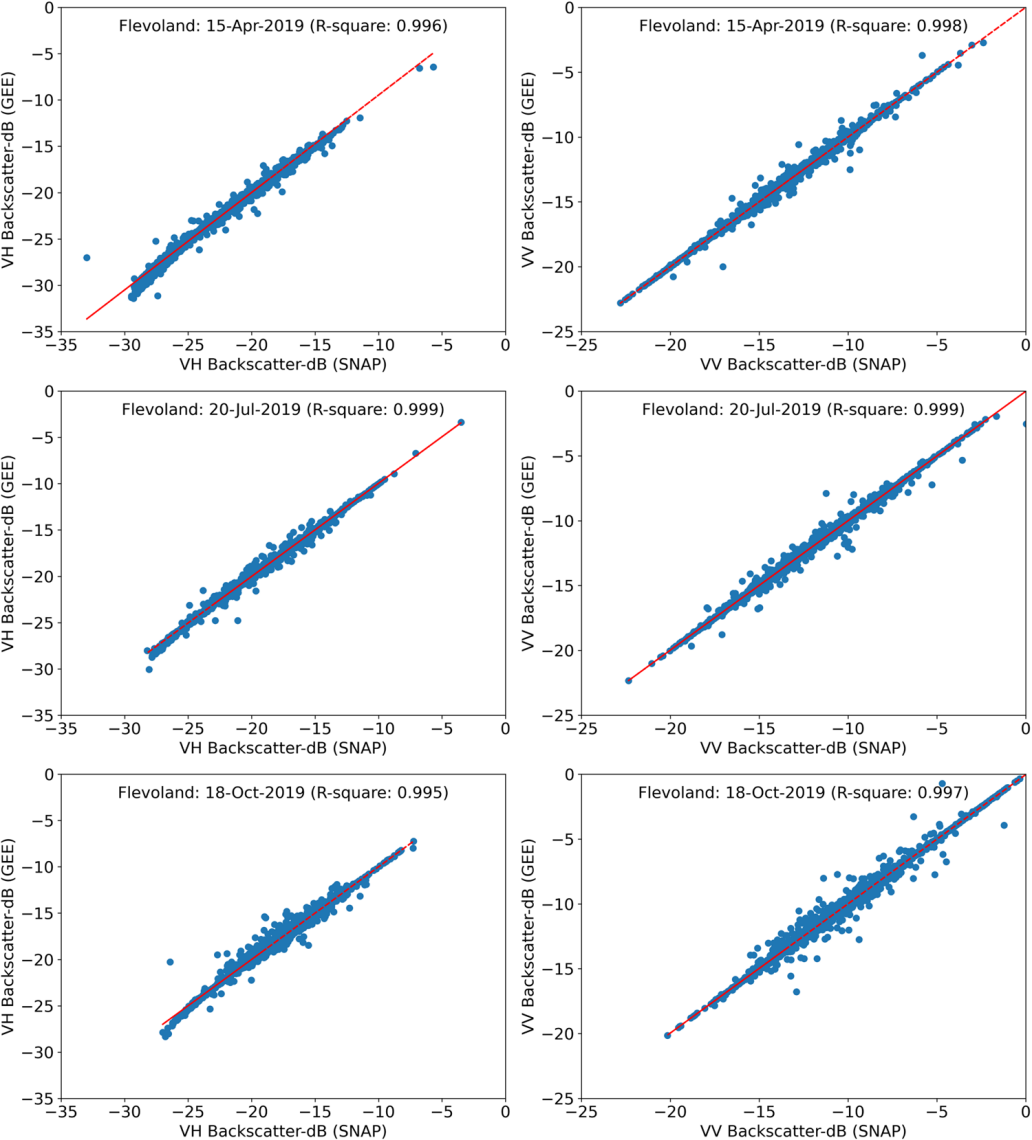
Comparison between GEE and SNAP extracted VH and VV backscatter values of Sentinel-1 GRD data acquired on 15 April 2019, 20 July 2019 and 18 October 2019 (RO-37) for BRP parcels in the Flevoland area. Every point in the scatter plots represents averaged value of one parcel.

The section describes the validation of the Agricultural SandboxNL database against the SNAP extracted Sentinel-1 backscatter values, and the flagging of the BRP parcels which are at the azimuth and range boundary of image geometry. The purpose of the validation is to control that no alteration of the data occurred during the data processing steps in GEE and python. The blue highlighted boxes in Fig. 1 indicate all processing steps to be validated. For validation, Sentinel-1 GRD data were separately downloaded from the Copernicus Open Access Hub^54^ and further pre-processed with the SNAP V8.0 toolbox^34^ to extract the backscatter values. The same processing steps were applied as in GEE to guarantee a sound comparison. The SNAP-processed backscatter output values were therefore considered as a reference.The validation was performed over the province of Flevoland, which consists of approximately 16,500 crop parcels. Sentinel-1 descending images in RO-37 were downloaded on 15-04-2019, 20-07-2019 and 18-10-2019 to span a typical summer crop season. Parcel conditions are generally heterogeneous in April, due to the bare soil surface and ongoing field preparation activities. July corresponds to the period with maximum crop cover and biomass. By October, most crops have been harvested. The GRD images processed in SNAP were imported to GEE in .tiff file format to apply the field buffer zones and to extract parcel-averaged VH, VV backscatter values. Finally, the validation was performed between the SandboxNL database and the SNAP toolbox processed images, by comparing the parcel-averaged backscatter values. The extracted backscatter values are highly correlated with those directly from SNAP, (Fig. 5) with a goodness of fit (R^2^) of at least 0.99 for all dates in both the polarizations. Despite the high R^2^ values, some deviations are visible between GEE and SNAP extracted backscatter. These deviations have already been reported in ESA SNAP toolbox community forum^58^. The reported difference in the R^2^ values between GEE and SNAP may be due to additional processing of the data within GEE (e.g. tiling approach).

### Data quality and flags

**Fig. 6:**
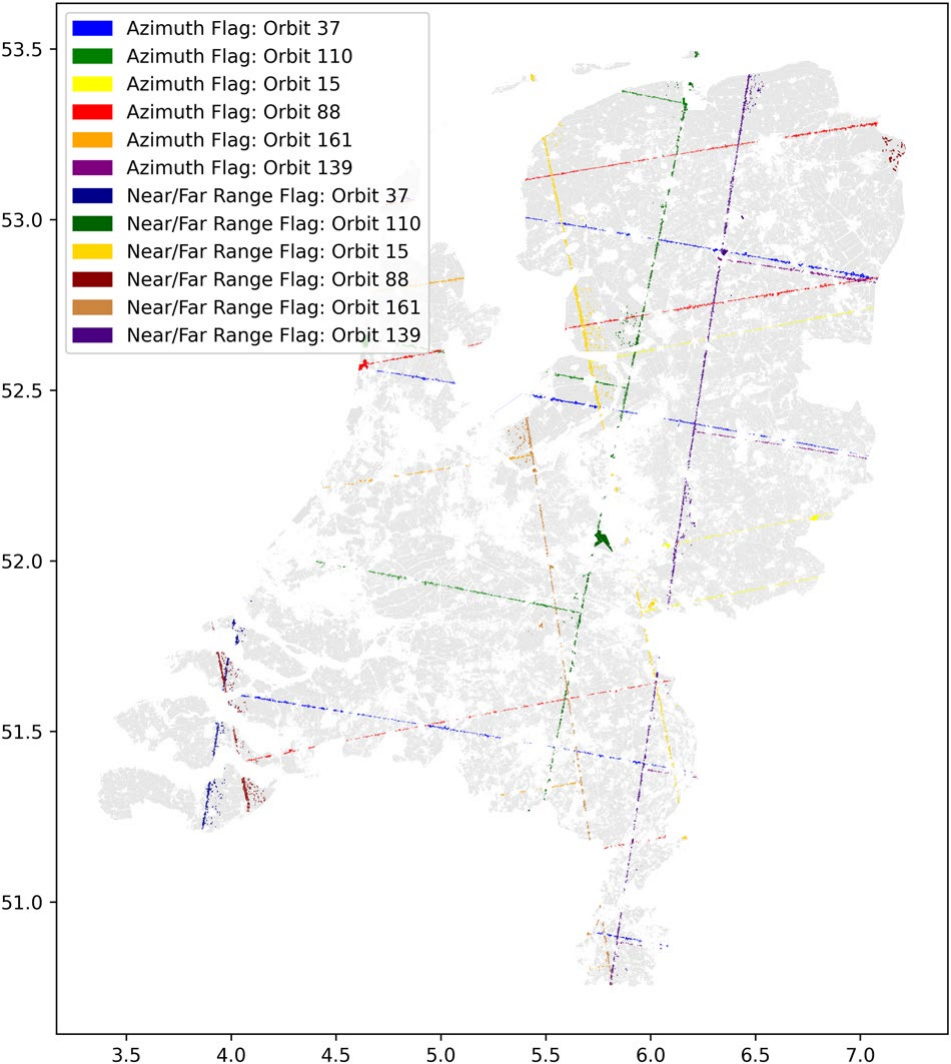
Flagged fields over the whole Netherlands for 6 RO covering the Netherlands (15, 37, 88, 110, 139 and 161) marking fields on the azimuth or near/far range image border.

Figure 2 shows that Sentinel-1 tracks may not be exactly covering the entire Netherlands administrative boundaries. In range direction, RO 38 and 88 cover a major part of the geographic extent of the Netherlands whereas RO 110, 139, 161 and 15 cover approximately half of the total geographic area. It is important to flag the parcels lying at the near and far range of the image borders in range direction as the pixel count in these parcel will vary depending on the relative orbit. The average backscatter and standard deviation were calculated when the parcel was completely within a Sentinel-1 image. On dates when the parcel was only partially covered, a data quality flag was assigned to parcels in the database as shown in Fig. 6.

The along-track dimensions of a data take obviously depend on the Sentinel-1 observation scenario that dictates that over Europe all passes are consistently acquired. The full along-track product from a single acquisition is segmented into slices with unique time stamps for data distribution^59^. In the SNAP toolbox, the *S-1 Slice Assembly* function seamlessly combines these sliced products into an assembled product without any data gap or discontinuity. However, this function is currently not available in GEE. In the Agricultural SandboxNL database generation, some crop parcels may be covered by two slices. To handle this continuity, the parcel average was obtained using a weighted average based on the pixel counts in each slice. The time-stamp of the slice containing most pixels was used as the data-stamp in the database. These azimuth border parcels were flagged as shown in Fig. 6.

In the processing chain every orbit was treated separately and the data from all orbits were concatenated at the end. The flagging methods compare the absolute difference between the unique list of dates and the actual list of dates. If the unique list of dates is smaller than the actual list of dates, the parcel gets flagged with 1, indicating that it is on the azimuth border of the image. Near and far range border effects have daily unique observations and but have different pixel counts. These filter conditions were applied on the fields at the outer edges of the image (<30.1 and >44.9 degrees of the approximate viewing incidence angle), and the parcels are flagged with a value of 2. The flag information was stored per orbit in the general parcel information.

**Fig. 7:**
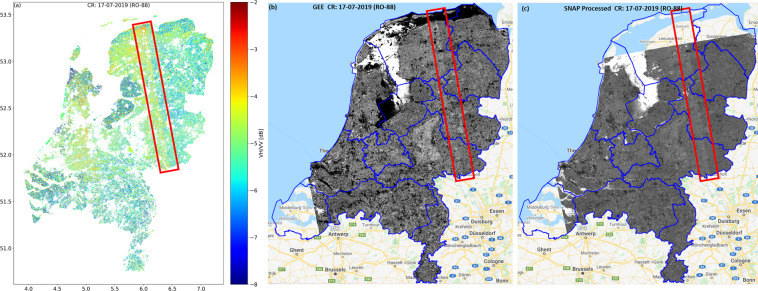
Figures illustrate interswath granularity due to thermal/system noise in VH/VV (CR) ratio in (**a**) Output database (**b**) GEE Ingested Sentinel-1 GRD image processed for CR calculation (**c**) SNAP processed Sentinel-1 GRD image processed for CR calculation. All images show the CR on the 17-07-2019 for RO 88. The blue outlines in Figure (**b**), (**c**) show the boundaries of the Dutch provinces. The red polygons highlight the jump in CR values observed for all cases.

Sentinel-1 acquires data in IW mode using the Terrain Observations with Progressive Scan (TOPS) multi-swath observation technique. After application of the thermal noise filter, some small radiometric jump could still be observed in some Sentinel-1 GRD images, especially at the subswath edges. This sometimes results in a visible discontinuity at the subswath edge in the generated database, particularly in VH-pol and cross-pol ratio images. One explanation for this undesired effect could be a small unresolved residual calibration bias in elevation that is further amplified when using VH/VV ratios. Figure 7 shows that the cross-pol ratio is influenced by this effect at interswath positions in all the shown images, i.e. generated database, GEE Image and SNAP extracted image of 17 July 2019 data in RO-88. Although in this particular case (mid July, well-developed crops), the backscatter at both co- and cross-polarization should only marginally be influenced by thermal noise, slight inconsistencies in the applied thermal noise removal procedure (equivalent in both GEE and SNAP) could also have led to such effect. Therefore, such residual calibration or processing bias is in the Sentinel-1 data products themselves, and not an artifact of the database generation. Nonetheless, the user should be mindful of this artifact when interpreting backscatter values in these areas.

## Usage Notes

The Agricultural SandboxNL database is freely available at the 4TU Centre for Research Data repository^57^ under a CC BY-SA 4.0 licence. The database was stored annually in *netCDF* format for the 12 provinces of the Netherlands. In addition, the re-projected and spatially buffered BRP parcel boundaries for the Netherlands for all the three years 2017, 2018 and 2019 are provided as shapefiles. In general, two types of information were stored annually: (1) Static Information and (2) Dynamic Information. Static information refers to the unique OBJECTID, parcel centroid, polygon, crop type and data flags which is updated every year, see subsection *Parcel data and attributes*0. Dynamic information consist of all data variables that change in time, i.e. VV, VH, VH/VV, relative orbit and local incidence angle. Depending on the needs of the users, data can be selected and filtered using either static or dynamic constraints. In the example *python* code, provided along with this publications repository, different scenarios are presented in which the data are visualized per longitude and latitude, crop type, OBJECTID, relative orbit and specific time stamp. The unique OBJECTIDs per parcel allow to join data across datasets. This gives users great flexibility to manipulate the data spatially and temporally to meet the their specific requirements. In this section, we demonstrate the usage of the Agricultural SandboxNL database to query and visualize data in space and time at national and province level using VH/VV cross-pol ratio. Additionally, Sentinel-1 SLC data derived coherence maps are shown over the Flevopolder region to showcase the InSAR coherence data included in the dataset.

**Fig. 8:**
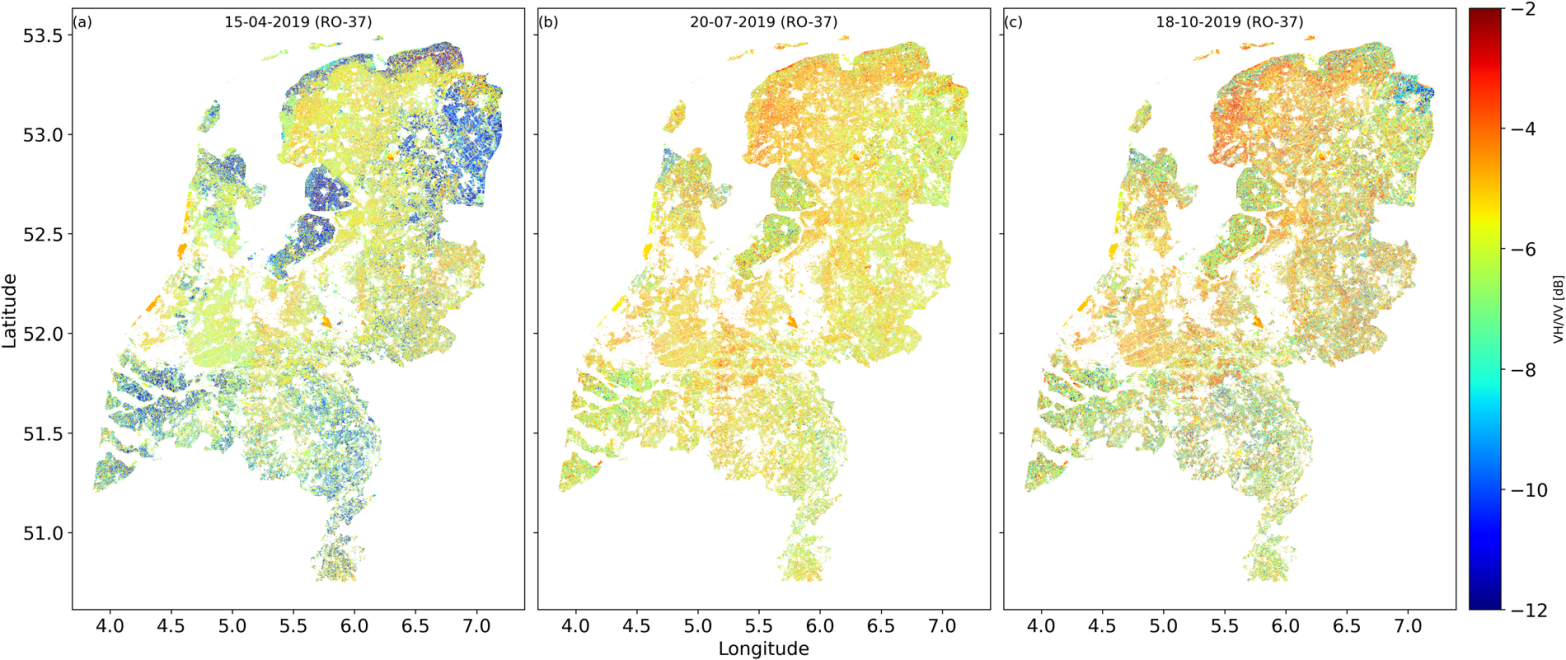
This Figure illustrates the spatial dynamics of VH/VV ratio for all the BRP parcels of the Netherlands for three dates (15-04-2019, 20-07-2019 and 18-10-2019) in RO 37.

In Fig. 8, an example is provided in which the database has been queried in time. It shows the parcel level cross ratio VH/VV (CR) over the entire Netherlands for three dates in 2019. Querying the data to extract an image of all of the Netherlands on dates before, during, and after the growing season allows the user to see that CR is clearly related to biomass^44,47^. CR values are low on 12 April 2019 compared to other dates as this the general crop planting time in the Netherlands. Provinces dominated by arable crop parcels such as Flevoland, Noord-Brabant, Drenthe, Limburg and Zeeland show CR values in the range of −12 to −8 dB as the soil is mostly bare and being prepared for planting. Most parcels in Friesland, Gelderland, Overijssel, Utrecht and Zuid-Holland are grassland (Perennial ryegrass). The CR values for the grasslands are in the range of −8 to −5 dB on 12 April 2019 data. Figure 8 shows that CR values on 20 July 2019 are higher than those on 12 April 2019 due to the peak vegetation biomass stage of the summer crops. For the summer crops such as maize, potato, onion, and barley, these values range from −8 to −5 dB due to strong scattering from the vegetation. Figure 8(b) on 18 October 2019, typically indicates the crop harvest period for summer crops in the Netherlands.

**Fig. 9:**
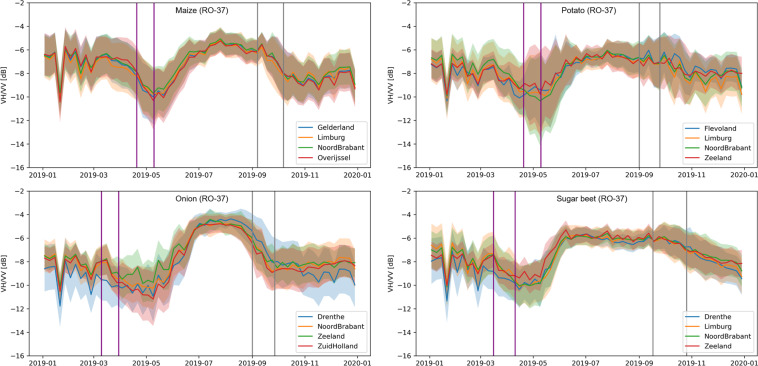
Time series of VH/VV ratio for all the maize, onion, potato, and sugar beet parcels in four provinces. The solid line indicates mean and shaded areas indicate standard deviation values in four different provinces selected according to the maximum no. of crop parcels. Purple and gray vertical bars indicate transplanting and harvest period, respectively.

In addition to querying the data by date to produce spatial maps, dynamic radar backscatter (VV, VH and VH/VV) information can be extracted to produce time series per crop type and per orbit. Data can be analyzed individually at parcel-level or aggregated using administrative boundaries. We have demonstrated one such case of querying the database used by extracting the cross-ratio values for four major summer crops i.e. maize, onion, potato, sugar beet. The CR values are extracted for the year 2019 in descending 37 RO and aggregated by province in Fig. 9. From the start of the year until the emergence, the Sentinel-1 backscatter observable (VV, VH and VH/VV) are mainly dependent on the attributes of exposed soil surface such as texture, roughness, moisture content and orientation^1^. The CR ratio decreases after March, probably due to changes in roughness. Standard deviation is high during the bare soil period due to differences in row orientation. The standard deviation in CR values for potato is much higher than the other crops due to the deep ridges in the potato fields. The CR values increase during the vegetative stages (after emergence) because VH is more sensitive than VV backscatter to the increasing biomass^44–47^. CR values start plateauing as the crop reaches maximum height for vertically oriented stalk crops and canopy coverage is maximum for broadleaf crops. The CR values start sharply decreasing from their peak at the ripening of corn and onion due to the reduction in vegetation water content and the start of harvesting. Unlike maize and onion, CR values for sugar beets decrease gradually as harvest may occur between mid-September to late November.

**Fig. 10:**
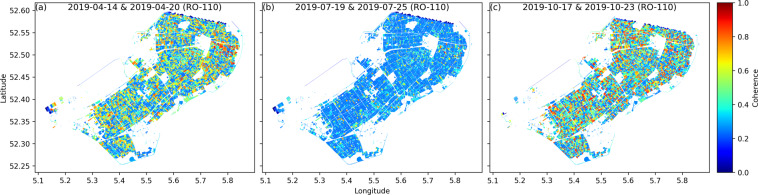
6 day parcel-level interferometric coherence (VV channel) of Flevopolder region of the Netherlands for three time intervals: (**a**) 2019-04-14 & 2019-04-20, (**b**) 2019-07-19 & 2019-07-25, (**c**) 2019-10-17 & 2019-10-23. Sentinel-1 SLC data in relative orbit 110 is used to generate coherence database.

**Fig. 11:**
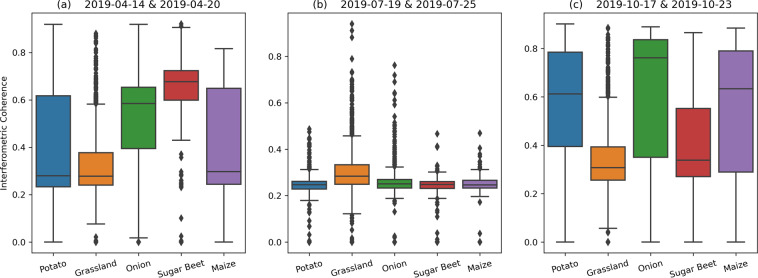
Box-plot of 6 day parcel-level interferometric coherence (VV channel, relative orbit 110) over the Flevopolder region considering five main crops (potato, grassland, onion, sugar beet and maize): (**a**) 2019-04-14 & 2019-04-20, (**b**) 2019-07-19 & 2019-07-25, (**c**) 2019-10-17 & 2019-10-23.

Figure 10 shows three samples of the interferometric coherence data included in the dataset. These images highlight the high coherence (>0.4) at the early transplanting stage and harvesting period when most of the backscatter response is from the exposed soil surface in VV polarization, and lower values (<0.3) during the vegetative phase of crop growth. These are due to crop growth, water content changes and motion of the canopy in the wind. In Fig. 11, the data from Fig. 10 are presented as box-plots, sorted by crop type. The mean coherence in grassland stays low throughout the year due to the permanent presence of vegetation cover. In April, higher coherence values are found in sugar beet and onion parcels compared to maize and potato. This may be because onion and sugar beet are planted, on average, one month earlier than maize and potato. The coherence is relatively high for potato, onion and maize parcels in October (Fig. 11(c)) as most parcels have been harvested. The lower values for sugar beet is due to the comparatively late harvest of sugar beets, which can extend beyond October.

This sensitivity of coherence for field practices such as plowing, seeding, tillage and harvesting is a known property, however not fully utilized for agricultural monitoring^45^. This allows the user to explore the potential value of transitions in interferometric coherence as indicators of crop emergence and harvest dates.

The ease with which users can download, manage and query the data in space, time and per system parameter means that this database is highly accessible for new users of SAR data. In addition, reducing the data volume from 1.6 TB per year to 4 GB, and providing processed data means that expertise in SAR processing software, and access to high-performance computing facilities is no longer a limiting factor for research and applications development. It is hoped that creating this capacity to explore and experiment with three years of SAR data for 770,000 agricultural parcels, on an almost daily basis, will stimulate the development of new techniques and applications of SAR data in agricultural applications.

## Data Availability

Python code to access, query, visualize and analyze the Agricultural SandboxNL database is distributed, with the dataset and accompanying documentation. GitHub repository to share all GEE/python scripts and information used to create Agricultural SandboxNL database. https://github.com/ManuelHuber-Github/Agricultural-SandboxNL.
